# The Financial Effect of the Electricity Price Forecasts’ Inaccuracy on a Hydro-Based Generation Company

**DOI:** 10.3390/en11082093

**Published:** 2018-08-11

**Authors:** Umut Ugurlu, Oktay Tas, Aycan Kaya, Ilkay Oksuz

**Affiliations:** 1 Management Engineering Department, Istanbul Technical University, Besiktas, Istanbul 34367, Turkey; 2 Industrial Engineering Department, Istanbul Technical University, Besiktas, Istanbul 34367, Turkey; 3 Biomedical Engineering Department, King’s College London, London SE1 7EU, UK

**Keywords:** profit loss, electricity price forecasting, mixed integer linear programming, self-scheduling, hydro-based generation company

## Abstract

Electricity price forecasting has a paramount effect on generation companies (GenCos) due to the scheduling of the electricity generation scheme according to electricity price forecasts. Inaccurate electricity price forecasts could cause important loss of profits to the suppliers. In this paper, the financial effect of inaccurate electricity price forecasts on a hydro-based GenCo is examined. Electricity price forecasts of five individual and four hybrid forecast models and the ex-post actual prices are used to schedule the hydro-based GenCo using Mixed Integer Linear Programming (MILP). The financial effect measures of profit loss, Economic Loss Index (ELI) and Price Forecast Disadvantage Index (PFDI), as well as Mean Absolute Error (MAE) of the models are used for comparison of the data from 24 weeks of the year. According to the results, a hybrid model, 50% Artificial Neural Network (ANN)–50% Long Short Term Memory (LSTM), has the best performance in terms of financial effect. Furthermore, the forecast performance evaluation methods, such as Mean Absolute Error (MAE), are not necessarily coherent with inaccurate electricity price forecasts’ financial effect measures.

## 1 Introduction

Electricity price forecasting has become an essential task since the liberalization of the electricity markets. It is integral for all the players in the energy markets, due to several reasons. Firstly, both supply and demand sides present their bids in the regulated markets according to electricity price forecasts. Secondly, bilateral contracts and energy derivatives also use longer term electricity price forecasts as reference points. Thirdly, large-in-scale demand side bidders, such as distribution companies, large industrial companies or pumped storage units can manage their purchasing behavior according to electricity price predictions. Last, but not least, generation companies (GenCos), such as hydro, natural gas and fuel oil can schedule their generation and bidding behavior according to the day-ahead price forecasts to maximize their profits.

This paper presents the influence of electricity price forecast accuracy on the profit maximization of GenCos. In particular, we use Mixed Integer Linear Programming (MILP) to schedule production strategies of a hydro-based power plant to minimize the profit loss of the companies. We make use of five individual and four hybrid forecast models to schedule the electricity production of the hydro-power plant. The main contributions of this paper are particularly:

- Extensive analysis of the financial influence of electricity price estimation inaccuracy;
- Analysis of statistical methods, Artificial Neural Networks (ANN), Long Short Term Memory (LSTM), Gated Recurrent Units (GRU) and hybrid methods for electricity price estimation;
- Use of a hybrid ANN–LSTM method for estimating electricity prices to maximize the profit;
- Detailed statistical analysis between electricity price estimation and profit maximization of GenCos.

### 1.1 Electricity Price Forecasting

Electricity price forecasting is an ever-improving research area and many different methods are implemented to forecast electricity prices. The review of Weron [1] categorizes electricity price forecasts into five main groups: (1) Multi-agent models, (2) fundamental models, (3) reduced-form methods, (4) statistical time-series methods, and (5) machine learning models. Generally, the first two groups are more applicable to the smaller markets. The first group includes game-theory type models for the smaller markets with less numbers of participants. Fundamental models require all supply and demand information to intersect both curves to obtain the price. Reduced-form methods are mainly successful in the price spikes, which are one of the important characteristics of electricity prices, and statistical methods include regression-type methods from relatively easy naïve methods [2] to complex models [3]. Machine learning methods include several different sub-categories, such as neural networks, fuzzy logic, support vector machines, etc. A time-dependent type of neural networks, recurrent neural networks, provide notably impressive results nowadays, especially with the addition of more than one layer, which is then called a deep neural network [4,5]. According to [5], deep neural networks, especially deep recurrent neural networks, such as Long-short Term Memory (LSTM) and Gated Recurrent Units (GRU), outperform the statistical time series methods like Seasonal Auto Regressive Integrated Moving Average (SARIMA), as well as shallow and deep Artificial Neural Networks (ANN). These findings are mainly in line with the results of [4]. Although LSTM and GRU, which are tailor-made for time series, are expected to perform better than deep ANN, Lago et al. [4] find out that deep ANNs are better than deep recurrent neural networks. On the other hand, a superiority of [4] to [5] is that it proposes 27 models and [4] can be used as a benchmark in the electricity price forecasting literature. Models of [4] also contain some deep hybrid methods, which motivated us to use the deep hybrid methods. Although current research has had promising results in favor of machine learning methods, Lasso regression applications [6,7], ensemble predictions [8–10], and hybrid works [11–14] also have successful results. One important example is the work of Chaabane [11], which combines SARIMA with Auto-Regressive Fractionally Integrated Moving Average (ARFIMA). However, as Aggarwal et al. [15] mentioned, still, none of the methods outperform the others regularly and continuously.

### 1.2 Generator Companies’ Profit Maximization

There are mainly two problems generation companies need to solve related to electricity price forecasting. The first problem is the self-scheduling of the power plants, which means the optimization of the production quantities for each hour of the next day according to the price forecasts. The second problem is presenting the correct price bids related to these quantities. This article will focus on the first problem. The purpose of this paper is to propose a Price Based Unit Commitment (PBUC) to a hydro-based GenCo according to the price forecasts of various methods, both from statistical and machine-learning methods, applied in [5]. According to the price forecasts, a GenCo will procure the production of electricity. It should be mentioned here that electricity is a non-storable commodity, whereas water can be stored. Thus, hydro GenCos have the opportunity of storing electricity in the shape of water, which eases most of the production costs and constraints compared to the other types of GenCos, i.e., thermal, wind or solar. In this sense, a self-scheduling optimization problem must be solved by using a technique, such as Mixed Integer Linear Programming (MILP), Lagrangian relaxation, dynamic programming or genetic algorithms [16]. There are two assumptions when optimizing the hydro-based GenCo’s self-scheduling [17]. The first assumption is that the GenCo is a price taker, which means that the price bids the GenCo presents do not have a significant effect on the determined market price. In a relatively big market, this assumption is easily justified, related to the capacity of the GenCo. The second assumption is that all the quantities offered will be accepted and sold. This assumption is also reasonable, considering that most of hydro GenCos, as well as solar and wind power plants, give their bids with zero prices and accept the clearing price.

In a pioneer work, Zareipour et al. [18] measure the economic impact of inaccurate electricity price forecasts from the demand side. Two different typical industrial loads, process-industry load and municipal water-plant load, are investigated, and the actual prices and inaccurate electricity price forecasts for the next 24-h are compared. One of the main findings of [18] is that Mean Absolute Percentage Error (MAPE) cannot reflect the economic value of improving the forecast accuracy. Therefore, other financial indicators should be checked to evaluate the financial effect of inaccurate electricity price forecasts. Another point is that the expectation from the forecast varies according to the type of customer. For example, the process industry needs accurate forecasts with respect to an exact threshold. On the other hand, for the water-plant, knowing the general trend of electricity prices over the planning period is quite helpful. Another important study, which was published right after [18], is Delarue et al. [17]. The main difference from [17] is that its point of view is mainly from the supplier side. Another distinction is that it uses MILP to solve the PBUC problem. In [17], four different power-plant types are examined. Combined cycle power-plant and pumped storage power plants are affected more by inaccurate forecasts than hydro and coal-fired classical thermal power plants, in terms of profit loss. Another interesting finding is that if inaccurate forecasts have an upside or downside bias, then profit loss gets affected by this bias as well. Downside bias, which means predicting the prices lower than the actual ones, cause higher profit losses. Mohammadi-Ivatloo et al. [19] also examine the economic impact of four different price forecasts compared to the actual prices for GenCos. [19] takes a hydro power-plant and a thermal power-plant into account. This research proposes two indices to evaluate the effect of inaccurate forecasts: The first one is the Economic Loss Index (ELI), which is the profit loss of the electricity price forecasting model, in terms of percentage, from the actual price profit; the second one is the Price Forecast Disadvantage Index (PFDI), which shows the profit loss per energy sold. According to the results of [19], traditional error measures do not always cause significantly high economic losses. This means that a model with lower forecast performance errors could cause higher economic losses than a model with higher forecast performance errors, and vice versa. Thus, according to Mohammadi-Ivatloo et al. [19], using ELI or PFDI instead of Mean Absolute Error (MAE) or MAPE as a financial effect evaluation method would yield less profit loss. In a related paper, Mathaba et al. [20] work on the same topic and propose a method which could be used in choosing the best forecast mechanism. According to Mathaba et al. [20], using the Rank Correlation (RC) method instead of Root Mean Square Error (RMSE) or MAPE would cause less profit loss. Mathaba et al. [20] use a coal-conveying system with storage, which allows the combining of the supply and demand sides. Three forecasting methods in the U.S. Pennsylvania-New Jersey-Maryland (PJM) market are performed. There are mainly three findings of [20]: Firstly, RC is a better indicator than RMSE and MAPE in terms of having less profit loss; secondly, price volatility, rather than mean price, has a higher effect on the profit loss. Therefore, models which take volatility into account could have less profit loss. Thirdly, profit loss is very dependent on the responsiveness of the load to electricity price changes. Research of Doostmohammadi et al. [21] proposes a completely different evaluation method for the same problem. First of all, it prepares a financial loss/gain (FLG) time series by using the real conditions of the electricity market. Secondly, they quantize this signal by using the Silhouette criterion and k-means clustering technique to simplify the problem. Then, the most informative variables are chosen from a feature selection problem by a combined technique. Lastly, by using all these methods and extreme learning machine, FLG predictions can be made which help the GenCos to optimize their scheduling. This is an interesting work because it combines the forecasting procedure with the profit loss calculation mechanism and the results show the positive effect of this evaluation.

To solve the self-scheduling optimization problem of the supplier company, different optimization methods are used in the literature. The most common technique is the MILP proposed in a pioneer work by Conejo et al. [22], which introduces a self-scheduling plan for a hydro producer in a pool-based electricity market. There are eight cascaded hydro power plants along a river basin in this system. It is a relatively big power plant, in terms of production capacity. Thus, it is difficult to fulfill the assumption that the supplier is a price taker and its bids do not change the market price. The objective of the optimization is maximizing the profit by selling electricity in the day-ahead market. For each plant, it takes nonlinear and non-concave three-dimensional relationships between the power produced, water discharged and the head of the related reservoir. Start-up costs are definitely in the calculation of profit. Similar to common techniques in the literature [23–25], Conejo et al. [22] also utilize the IEEE 118-bus test system [26] as the hydro power plant. Applied to the Spanish day-ahead market prices in 2001, daily profits are around 600,000$, which shows the great capacity of the hydro power-plant system. Esmaeily et al. [23] add some practical constraints, such as valve-loading cost, dynamic ramp rate and prohibited operation zones. As in [22], they takes the price forecast errors to understand the price uncertainty. They also performs a Lattice Monte Carlo Simulation for the effects of spinning and non-spinning reserve prices. Working hours of the hydro power-plant correspond to the high price hours in this model, which cause relatively high profits. Bisanovic et al. [27] is another study about the hydro-thermal self-scheduling problem in the day-ahead electricity market. As in the previous papers, Bisanovic et al. [27] use MILP, because this optimization method allows one to have non-convex and non-linear items as constraints. The difference for [27] is that it takes the long-term bilateral contracts, in addition to the hourly day-ahead electricity price forecasts, into account. As another difference, the system in this research is the combination of the thermal and hydro power plants. On the other hand, a common point with the other papers [16,22,23] is that this model also utilizes the piece-wise linear model to represent the non-linear functions. [27] solves the PBUC problem for four different types of plants: Thermal, combined cycle, cascaded hydro and pumped storage. This paper also utilizes the IEEE 118-bus system and applies mixed integer programming (MIP), which is compared with the Lagrangian relaxation (LR) method. Yamin and Shahidehpour [28] utilized transmission congestion and locational marginal prices as well as fuel and emission constraints in their model. Shahidepour et al. [29] give a broad overview in the forecasting, scheduling and risk management of power systems. With the availability of high memory and greater computational power, MIP and MILP type optimization techniques have become state-of-the-art methods for PBUC problems.

### 1.3 Turkish Market

Although there are some works [5,30–32] on electricity price forecasting in the Turkish electricity market, the financial effect of electricity price forecasts’ inaccuracy has not been investigated. Due to the nature of the Turkish market, with many zeros similar to the Spanish market [33], and an increasing renewable share similar to the German market [13], the Turkish market needs investigation, and can give some insight about the other Southeast Europe markets [34] in addition to Spanish and German markets. The Turkish market is an emerging market with very specific features as discussed in [32,35,36]. Mainly due to the country’s climate, in the summer months, electricity consumption and the prices are almost as high as the winter months. Turkey has an inter-connected grid with Greece and Bulgaria, but the share of the import and export electricity never exceeds 1% of the daily consumption. One of the most important features of the Turkish market is the high share of hydro energy of 34.2% and the increasing share of the wind energy of 7.6% at the end of 2016, in terms of the installed capacity [37]. Due to the snow-melt effect in the spring months and the wind effect in spring and autumn months especially, prices tend to decrease in these seasons. Turkish electricity prices are limited from 0 to 2000 TL/MWh, which is approximately 562 $/MWh by the 2016 average exchange rate [5]. This rule does not allow the Turkish market to have negative prices, which is similar to the Canadian market [38] and the opposite of the German market [13,39]. Taking the number of zeros in the Turkish market into account, we can mention that it has a negative effect on the market efficiency. However, on the other hand, the price cap of approximately 562 $/MWh is very high and has never been reached in the short history of the market since December 2011. However, prices tend to have very high values, especially in the lack of natural gas for the power plants. In the near future, two nuclear power plants will be established as the first attempts of Turkey; solar power will be integrated into the grid; wind share still has an increasing trend, but the subsidies on the wind power plants will stop in two years. As another point of interest, intra-day market and energy derivatives are also developing and in need of research. To sum up, there is a rapidly changing and improving environment in this major emerging market, which motivates our work to investigate the specific characteristics of this market. Evaluating the effects of electricity price forecasts’ inaccuracy in the Turkish Day-Ahead Market and comparing it with the results of the other markets could help us to understand the nature of the market better.

In addition to this, our paper is the first one which takes deep neural networks, especially deep recurrent neural networks, as forecast methods to investigate the relationship between the forecasts’ inaccuracy and the financial effect caused by this inaccuracy. Moreover, it also combines the predicted electricity prices of these models as hybrid methods and compares them with the individual counterparts, in terms of profit loss.

The remainder of the paper will be structured as follows: Section 2 describes the dataset and the method. In particular, electricity price forecasting, Price Based Unit Commitment (PBUC) and the financial effect measures are detailed. In Section 3, results are given and discussed. Section 4 concludes the paper and investigates some further research ideas.

## 2 Data and Methods

We use data from mainly two sources in our simulations. Firstly, Turkish Day-ahead Market electricity prices are taken from EPIAS [40] between 2013 and 2016. Secondly, IEEE 118-bus-system test data [26] are used in the hydro-based GenCo’s self-scheduling.

**Figure 1:**
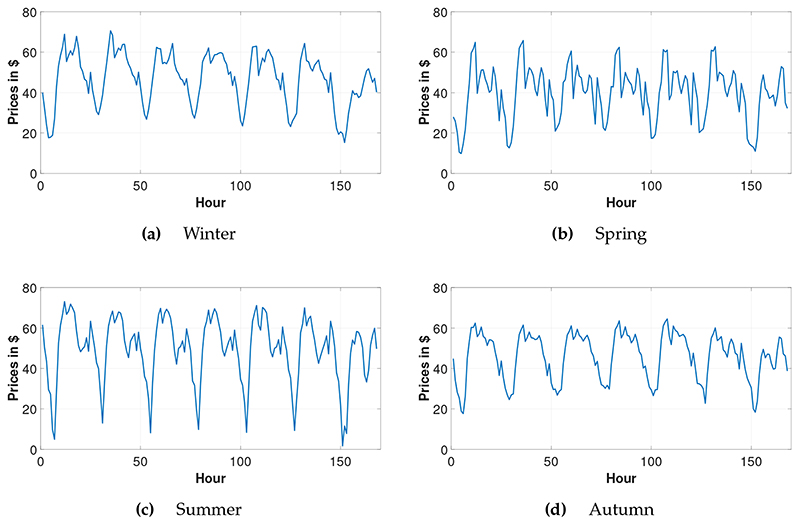
Hourly averaged electricity prices for each season of 2016.

The seasonal average of each hour of the week from the year 2016 is visualized in Figure 1. We have listed the descriptive statistics of the test data for each hour of the data in the year 2016 in Table 1. Firstly, we need to mention the intra-day seasonality of the data. The price average at 6 am is nearly one-third of the price average of 11 am. Early morning hours have the lowest prices with the highest variation. It is especially difficult to forecast these early morning prices. Secondly, there are many zeros in the prices, which make the preliminary studies of the data difficult. In the statistical methods, to make the data stationary, there is a need for transformation. Due to these low prices, it is impossible to take the logarithmic returns. Moreover, prices around zero cause biased results in the MAPE numbers. Thirdly, the highest price of 2016 is 132.36 $/MWh, which is beyond *μ* + *5σ* for 10 am. Figure 1 mainly visualizes the intra-year seasonality. Although the range is very small in the spring and autumn months, in the summer the average daily range of the prices are as high as 70 $/MWh. Figure 1c illustrates the average summer prices of 2016. Due to religious holidays, which are not coherent with the Europe, prices do not show co-movement in Europe’s and Turkey’s holidays. The prices show a sharper decrease on Fridays at lunchtime due to the Friday prayer, which can be seen especially in Figure 1a. Another difference to the European market is the half-day working habit on Saturdays. In Figure 1, relatively high prices can be observed in the morning hours on Saturdays. Due to the usage of the air-conditioning because of the hot climate in the summer months, prices are very high in the day-time. On the other hand, prices in the early morning hours are very low. This causes lots of spikes, which makes electricity price forecasting especially difficult in the summer months. In Figure 1b, due to the snow-melt effect, hydro power-plants work in high levels and produce relatively low-priced electricity.

### 2.1 Electricity Price Forecasts

Electricity prices are forecasted for 6 weeks of every season in 2016 by using various methods following [5]. A 3-year rolling window scheme is used for the estimation and 24-step ahead hourly forecasts are done by using the endogenous variables, which are the 1st, 24th, 48th and 168th lags of the price series. In this way, forecasts are done for 2 weeks from all the months of 2016. Five different forecast methods, namely the Naïve method, SARIMA, ANN, LSTM, and GRU are utilized in this paper. In addition to these models, four different combinations of the best performing ANN, LSTM and GRU models’ forecasts are also used to evaluate the financial effect of the forecast inaccuracies compared to the “best” ex-post actual prices case. This paper uses the models from [5]; brief descriptions of all the models are given below in the related sections.

#### 2.1.1 Naive Method

The naïve method is a benchmark in the electricity price forecasting literature, which takes the previous day’s or previous week’s same hour as the forecast price [2]. According to [2,41], unsuccessful forecasts cannot outperform this benchmark method. The naïve method is described as:

(1) $$P_{d,h}=\{\begin{array}{c} P_{d-7,h}+\epsilon_{d,h},\;\text{Monday, Saturday, Sunday }\\ P_{d-1,h}+\epsilon_{d,h},\;\text{Tuesday, Wednesday, Thursday, Friday } \end{array}$$

P*d,h* states the price of the selected day and hour. ϵ*d,h* stands for the noise term.

#### 2.1.2 Seasonal Auto-Regressive Integrated Moving Average (SARIMA) Model

ARIMA is a special kind of regression, which takes the past prices (AR), previous values of the noise (MA) and the integration level (I) of the price series into account. In SARIMA, a seasonal component (S) is also involved in the estimation process. Generally only the intra-weekly nature of the series is incorporated as a seasonal component, but in the electricity price series it is required to deal with the intra-day and intra-year seasonality as well. Therefore, the triple SARIMA model [42] is performed by maximum likelihood assuming Gauss-Newton optimization. Equation (2) refers to the triple SARIMA model.

(2) $$\phi_{p}(L){\text{Φ}}_{p_{1}}({\text{Ł}}^{s_{1}}){\text{Ω}}_{P_{2}}({\text{Ł}}^{{\text{S}}_{2}}){\text{Γ}}_{P_{3}}({\text{Ł}}^{{\text{S}}_{3}})(y_{t}-a-bt)=\theta_{q}(L){\text{Θ}}_{Q_{1}}({\text{Ł}}^{s_{1}}){\text{Ψ}}_{Q_{2}}({\text{Ł}}^{{\text{s}}_{2}}){\text{Λ}}_{Q_{3}}({\text{Ł}}^{{\text{S}}_{3}})\epsilon_{t}$$

*y_t_* is the load in period *t*, *a* is a constant term, *b* is the coefficient of the linear deterministic trend term, *ϵ_t_* is a white noise error term, Ł is the lag operator, and *φ_p_*, Φ_p1_, Ω_P2_, Γ_P3_, θ*_q_*, Θ_*Q*1_,Ψ_Q2_, Λ_Q3_ are the polynomial functions of orders *p*, *p*_1_, *P*_2_, *P*_3_, *q*, *Q*_1_, *Q*_2_ and *Q*_3_, respectively [5,42].

Our triple SARIMA model can be stated as (1,0,1)_1_ *x*(1,0,1)_24_ *x*(1,0,1)_168_. To comply with the other statistical methods, an ARMA(48,48) component is also added to this model.

#### 2.1.3 Artifical Neural Networks

There is a growing interest in Artificial Neural Networks (ANN) in the electricity price forecasting literature [43–45] as well as many other areas. ANN consists of layers of neurons, which are connected densely. They are also called Multi-layer Perceptrons (MLP). In this paper, we use three-layer ANN, where each layer has 10 neurons and a final layer estimates the forecast values. The batch-size is 3-years during training, the learning rate is 0.001, the momentum of the optimizer is 0.90 and 300 epochs are used [5].

#### 2.1.4 Long Short Term Memory

Long short term memory (LSTM) is a type of the recurrent neural networks (RNNs). RNNs are the best fit for the time-dependent problems, because they allow the information to persist, with their loops-allowing architecture. Due to their nature, which allows using the temporal information as the input, RNNs are the best models for the time series data. In a unique type of the recurrent neural network, LSTM, each node can be used as a memory cell, which can store the information from the other cells as well. Therefore, LSTM addresses the vanishing gradients problem of the previous time steps. The input, forget and output gates of LSTM control the existing memory and take the information from the first moments of the learning process and use it much later. This feature gives the opportunity to modeling long-term dependencies. The same batch-size, learning rate, momentum of the optimizer and epochs with the ANN model are used for the 3-layer LSTM model [5].

#### 2.1.5 Gated Recurrent Units

Gated recurrent units (GRU) are another type of RNN, which is utilized in time-dependent problems with considerable success. GRU consists of two gates, namely the reset gate and update gate. The update gate determines how much of the previous memory will be used and the reset gate decides how to combine the previous memory and the new input. The main aim of the GRU is very similar to LSTM, which is taking long-term dependencies into account. However, in GRU there are only two gates and fewer parameters than LSTM. Instead of having only a reset gate, as in LSTM, in GRU there is both a reset gate and update gate. Another difference is that LSTM has output gates, but GRU does not have any. In our experiment, we use a 3-layer GRU model with the same features of the ANN and the LSTM model [5].

#### 2.1.6 Hybrid Models

We also form four different hybrid models by averaging the forecasts:

1. 50% LSTM–50% GRU
2. 50% ANN–50% GRU
3. 50% ANN–50% LSTM
4. 33% ANN–33% LSTM–33% GRU

The combinations are selected according to the best performing models in [5]. We examine the performance of the hybrid models, consisting of neural networks, and compare them with the individual model counterparts in terms of the financial effect of the forecast inaccuracy. This is the first work which investigates the hybrid models from this point of view.

### 2.2 Hydro-Based Power Plant

In order to model the behavior of GenCos, we used IEEE 118-bus-system test data [26]. These data are from a hydro-based power plant with eight cascaded units, which is a state of the art data set in the literature [22,23]. Although this data give much information about the units, topology, start-up costs, reservoir levels etc., they are for a massive system, which has the generation capacity of approximately double of the biggest hydro power plant in Turkey. Therefore, it is impossible to assume that this hydro power plant will work as a price taker in the Turkish market without affecting the market prices. For this reason, a modified version of these data are used and only the first two cascaded units are taken into account. The goal function, costs and the constraints are given to the model in General Algebraic Modeling System (GAMS) software. The PBUC problem is solved for the production amounts of each hour of the forecast days by MILP, which allows the hydro-power plant to self-schedule. Production amounts for each hour of the day are calculated for all the mentioned models: Actual prices, five different models and four hybrid models. This process is repeated for the predicted 168 days of 24 weeks. Then, the production amounts are multiplied by the ex-post actual prices to calculate the revenue. Lastly, costs are subtracted from the revenue and the profits are obtained.

Price Based Unit Commitment According to Mixed Integer Linear Programming

In this study, we used a Mixed Integer Linear Programming (MILP) model adapted from [22,23] to solve the self-scheduling problem of the hydro GenCo. Conejo et al. [22] represented a set of non-concave and non-linear performance curves showing the relationship between the reservoir head, the water discharged and the power output. They used piece-wise linearization to deal with these non-concavities and non-linearities of the performance curves, and proposed a mixed integer linear programming model. In this study, we use the same mathematical model by adding only one constraint related to maximum spillage amount adapted from [23]. The formulation of the mathematical model can be found in Appendix A.

### 2.3 Financial Effect of the Forecast Inaccuracy Measures

Having these production amounts and the related costs gives us the opportunity of calculating the actual profits for each model. The accurate prediction of the actual prices should translate to maximum profit during the sale of electricity. The difference between the profit of the forecast model and profit of the ex-post actual price model is called profit loss [17]. For choosing the best performing model, Mean Absolute Error (MAE) or Mean Absolute Percentage Error (MAPE) are the most common methods. We prefer to use MAE instead of MAPE because of the reason that MAPE values are biased with the actual electricity price values, which are around zero. Previous literature [19,20] suggest that there is a discrepancy between the general forecast model decision methods and the profit loss models. Therefore, Economic Loss Index (ELI) and Price Forecast Disadvantage Index (PFDI) [19] are also used to measure the financial effect of forecast inaccuracy. These measures are described as follows:

(3) $$\text{ELI }=\frac{{\text{ Profit }}_{\text{actual }}-{\text{ Profit }}_{\text{forecast }}}{|\text{ Profit actual }|}$$

(4) $$\text{PFDI}=\frac{{\text{ Profit }}_{\text{actual }}-{\text{ Profit }}_{\text{forecast }}}{\sum_{t=1}^{T}E_{t}}$$

where, *∑_*t*=1_^T^* E_t_ is the total energy sold in the market, in terms of MWh.

ELI demonstrates the profit loss as a percentage due to the inaccuracy of the forecast. Although it is not the case in our experiment, actual profit can be negative due to the ramp-up prices, constraints and limits. Thus, an absolute value of the actual profit is used in the denominator. Another point is that negative ELI is also possible due to unexpected higher profits of the forecast model than the ex-post actual prices case. PFDI is another financial effect measure, which calculates the profit loss per energy sold in terms of $/MWh. It must be mentioned that these models do not show the accuracy of the forecast, but the financial effect of forecast inaccuracy [19].

## 3 Results and Discussion

In this section, we report the profit loss comparisons in relation to electricity price forecast accuracy. In Section 3.1 we report profits obtained from all the methods, including ex-post actual prices: Profit losses, Economic Loss Index (ELI), Price Forecast Disadvantage Index (PFDI) and Mean Absolute Error (MAE) of the forecast methods for 24 weeks, two weeks from each month. Then, in Section 3.2, we compare the seasonal performance of the methods in terms of profit loss. In Section 3.3, we visually show the relationship between MAE and ELI for each hour or the day. We also illustrate the energy price profile and production schedule of the power plant for an exemplary day. This allows us to measure the inaccurate forecasts’ financial effect on the hydro-based power plant. Finally, we evaluate the statistical significance in Section 3.4 and discuss the impact of the results.

### 3.1 Profit Loss Comparison

This section demonstrates profit, profit loss, ELI, PFDI and MAE results for the hydro-based GenCo’s self-scheduling scheme according to various forecast methods, in addition to the ex-post day-ahead electricity prices. Table 2 gives the results as the total of the 24 weeks, six weeks from each season and best results are highlighted in the table. According to our results, ANN–LSTM is the best method in terms of financial effect measures. Scheduling the GenCo according to ANN–LSTM method would cause a profit loss of $216,410, 2.20% ELI and 1.1856 PFDI, compared to the ex-post actual prices scheduling. On the other hand, ANN is the best-performer by a small margin in terms of forecast performance measure MAE. This shows us that the forecast performance measures and the financial effect measures are not necessarily coherent with each other. Moreover, other hybrid methods ANN–LSTM–GRU and LSTM–GRU are the second and third best performing models, respectively, according to profit loss.

### 3.2 Seasonal Performance Comparison

**Figure 2:**
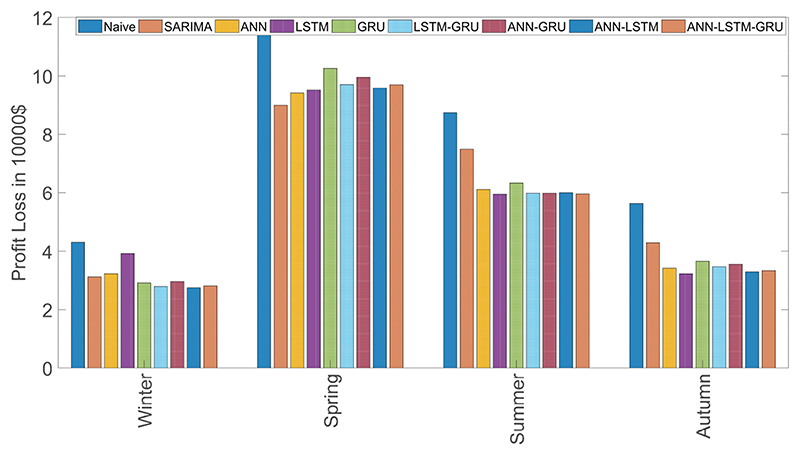
Seasonal profit loss results.

Figure 2 demonstrates the profit loss of the hydro-based GenCo’s self-scheduling scheme according to various forecast methods divided into seasons. We report the average results for six weeks of each season. First of all, we investigate the variation of the profit loss levels according to the seasons of the year. In the examined period, profit loss levels of winter and autumn are relatively small. On the other hand, profit losses are very high, especially spring, at the level of $100,000. Although ANN–LSTM is the best model only in winter, we observe the stable performance of the hybrid models. On the contrary, the performance of the individual models are not very stable. Relatively good performance of LSTM is shadowed by the poor performance in winter.

### 3.3 Energy Price Profile and Production Scheduling

**Figure 3:**
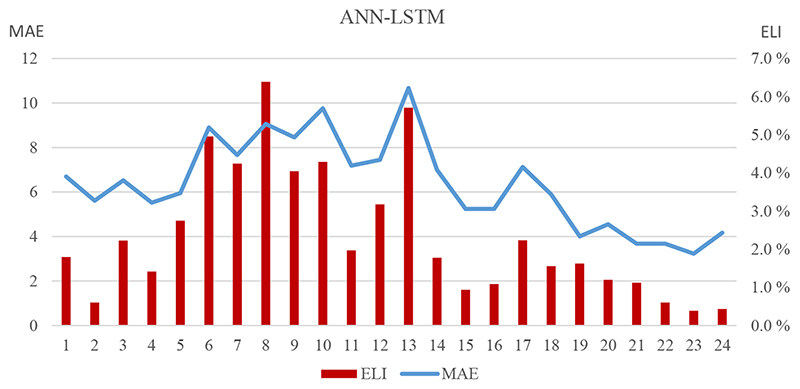
MAE and ELI Results of the ANN-LSTM Model for 24 Forecast Weeks.

Figure 3 reveals the relationship between MAE and ELI according to the LSTM model’s forecasts and the related scheduling of the GenCo. It must be mentioned that these values are from the 24 weeks of 2016, and, therefore, they are not continuous values. However, they give information about the co-movement of MAE and ELI. Although we observe the co-movement of MAE and ELI in general, on the last days, MAE levels do not follow the decreasing trend in the ELI numbers. This is further evidence for a difference between the forecast evaluation measures, such as MAE, MAPE, RMSE and the financial effect measures, such as profit loss, ELI and PFDI.

**Figure 4:**
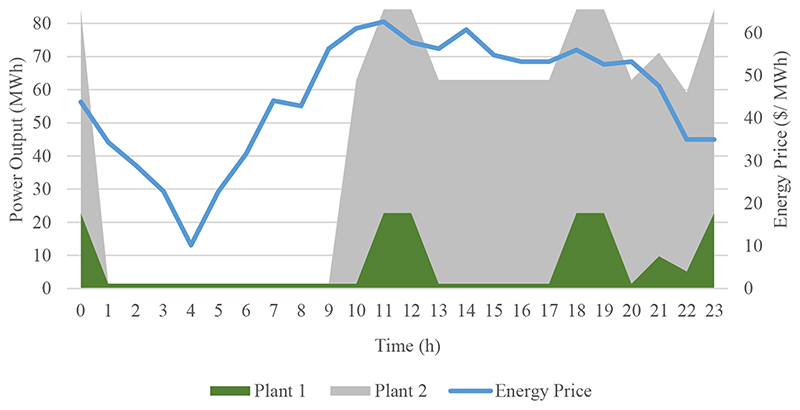
Energy Price Profile and Production Schedule of Plant 1 and 2 based on ANN-LSTM Method on 14 November.

**Figure 5:**
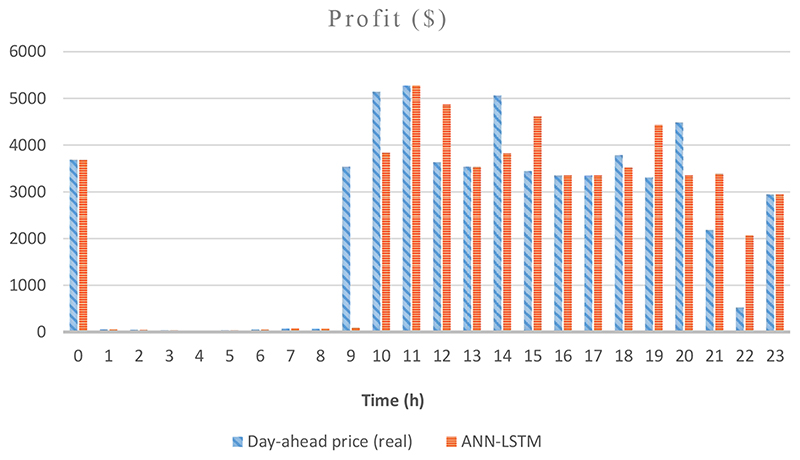
Profit of ANN-LSTM Model and Ex-post Day-ahead Prices According to the Hours of the Day on 14 November.

Figures 4 and 5 are the graphs for the scheduling and profits of the GenCo on a randomly chosen day, 14 November. Figure 4 demonstrates the relative success of the ANN– XLSTM model in the scheduling of the hydro power plant by showing the energy prices and power output of both units. It is observed that the power plant does not work in the lower price zone during early morning hours, and both units work in the maximum capacity in the peak-price hours. Due to the ramp-up costs, first, the greater unit, Plant 2, starts to operate, and in the higher demand moments Plant 1 gets activated as well. As a negative point, we can mention the 1 h delay in the production at 9. Figure 5, which shows the profits according to ex-post day-ahead scheduling and ANN-LSTM scheduling comparatively, supports this point by showing the profit of the ex-post day-ahead scheduling compared to almost no profit of ANN-LSTM scheduling at 9. Furthermore, ANN–LSTM scheduling produces more electricity and makes a profit in relatively low price levels at 21 and 22. On this day, scheduling according to ex-post day-ahead price forecasts resulted in $57,223.38 profit compared to the LSTM model scheduling, which caused $56,097.34 profit. It means $1126.04 profit loss for the ANN–LSTM model in one day for a relatively small power plant, which has only approximately 1089 MW production on this day.

### 3.4 Diebold-Mariano Tests

**Figure 6:**
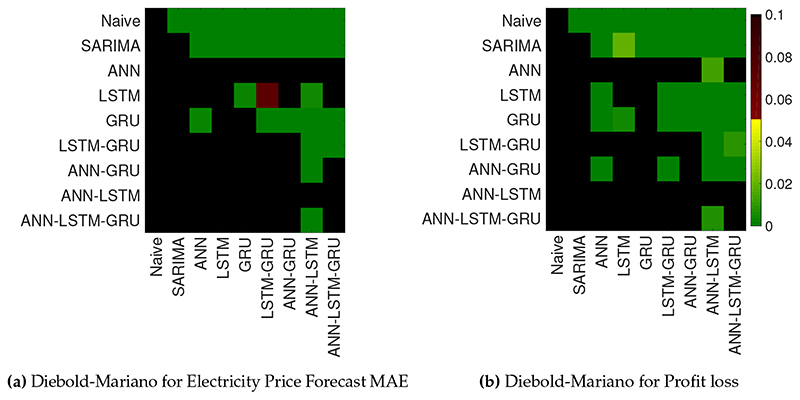
Results of the Diebold-Mariano tests defined by the MAE values and profit loss differential series in between different models. The figure indicates the statistical significance (green) for which the forecasts of a model on the X-axis are significantly better than those of a model on the Y-axis. The statistical significance of the difference between the MAE values does not translate fully into a difference in Profit loss.

The Table 2 can be used to provide a ranking of the various methods, however no statistically significant conclusions can be drawn on the performance of the forecasts. To showcase the statistical significance of the performance difference between all model variations, we use a Diebold-Mariano test [46], which takes the correlation structure into account. In Figure 6a, we show the *p*-values for the Diebold-Mariano tests between hybrid methods, neural networks-based methods and the statistical methods for 4032 h of the 24 weeks we investigated. In Figure 6b, we show the statistical significance test in between the MAE values and profit losses for each method to illustrate the difference between the MAE and profit loss.

Figure 6a demonstrates the successful performance of the ANN model and the ANN–LSTM model, in terms of MAE. However, there is not a significant difference between both models and it is not possible to choose one model over another. However, according to the profit loss values, ANN-LSTM model significantly outperforms all the other models, including ANN. It is evident that forecast performance measures, such as MAE and the financial effect measures, such as profit loss give different results, in statistically significant terms.

## 4 Conclusions

In this paper, we propose self-scheduling schemes according to nine different forecast methods by using Mixed Integer Linear Programming for a relatively small hydro-based GenCo with approximately 1 GWh production per day. Nine forecast schemes include a benchmark naive method, a statistical triple SARIMA model, machine learning ANN, LSTM and GRU models, in addition to the hybrid methods by the combination of the machine learning models. Additionally, we utilized ex-post actual prices as the perfect prices to schedule the power plant for optimum performance. This allowed us to calculate the profit loss, ELI, and PFDI as the financial effect measures. We also compare the relationship between the financial effect measures and the forecast performance evaluation measure, namely MAE.

This is the first paper which explores the use of hybrid methods from the financial effect of the forecast inaccuracies point of view. According to our results, ANN–LSTM model is the best performing one in statistically significant terms. Moreover, other hybrid methods ANN–LSTM–GRU and LSTM–GRU are the second and third best models, respectively. On the other hand, as individual models, ANN performs relatively well, but especially naive method, SARIMA and GRU would cause higher losses to the generation company. As the literature [11,12] suggest, the usage of hybrid works and the combination of the price forecasts [14,47], we also found out the statistically significant superiority of the hybrid ANN–LSTM method. Our findings are also in line with the works of [4,48], which advocate the use of hybrid methods in deep learning electricity price forecasting applications.

Another finding of this paper is that it supports the conflict between the forecast performance evaluation measures, such as MAE, and the inaccurate electricity price forecasts’ financial effectmeasures, such as profit loss, ELI, and PFDI. Even though the ANN is the best method in terms of MAE, and there is not a statistically significant difference between the ANN and ANN–LSTM methods according to the forecast errors; in terms of financial effect measures, ANN–LSTM is better than all the other methods, including ANN. Although the general trend is the same for MAE and financial effect measures, ELI in Figure 3, there are some conflicting weeks, which cause this discrepancy. Our results support the findings of [18–20] in the Canada and U.S. markets on this conflict; in a smaller power plant, in a different market and with various new forecast methods from [5] and the combinations of these forecasts.

As Figure 2 illustrates, the seasonality influences financial effect measures. Even though LSTM is successful in spring, summer and autumn; the poor performance in winter affects the success and reliability of this method. On the other hand, hybrid methods, especially ANN-LSTM, give stable and reliable results. For instance, SARIMA model shows a very good performance in spring. It could be helpful to change the models according to time periods. This is an avenue of improvement for our work.

In this paper, we focus on the relation between electricity price forecasting accuracy and profit maximization. For a fair comparison between different methods, we do not focus on the variable selection and just compare the methods with the same input variables. We compared five forecast techniques and four combinations of these techniques. In all the models we used the same endogenous variables; 1st, 24th, 48th, and 168th lags of the prices to compare the effects of the various models. In [5], it is observed that machine learning models outperformed the triple SARIMA and the other statistical models. In our current experimental setup, statistical methods, such as SARIMA, and naive method do not perform well. However, by using more complex statistical methods [3], different results could be obtained. We appreciate the hybrid models [11,12,49], dimension reduction techniques [6,7] and the automated variable selection works [50], which utilize a variety of variables and choose the best ones. An obvious further research topic is using the available input variables to the the fullest and evaluate the relation between electricity price forecast accuracy and profit maximization.

This paper opens the path for further research on the relation between electricity price prediction accuracy and profit maximization. Firstly, hybrid models show impressive potential and can be instrumental techniques for profit maximization. Secondly, the best performing models vary according to the test period. It could give the opportunity of using different models in different periods. Further research on this issue has the potential to minimize the profit loss of the suppliers. Thirdly, applying a non-linear programming technique for the PBUC could increase the scheduling performance. Lastly, solving the same problem for other types of suppliers, more markets and different time periods would check the generalizability, robustness and accuracy of these results.

## Supplementary Material

Appendix

## Figures and Tables

**Table 1:** Descriptive Statistics of the Turkish Day-Ahead Electricity Prices (Dollars/MWh) According to the Hours of the Day.

Hours	Mean	Standard Deviation	Lower Bound	Upper Bound	Median
0	49.27	13.47	0.28	78.42	47.98
1	41.80	14.99	0.00	78.05	42.84
2	35.89	15.98	0.00	76.69	37.85
3	27.40	16.31	0.00	76.13	27.57
4	25.42	16.55	0.00	76.12	26.27
5	23.77	15.36	0.00	77.86	25.12
6	22.65	17.62	0.00	77.94	24.55
7	34.52	17.54	0.00	78.08	40.27
8	44.76	17.91	0.00	79.20	48.88
9	55.74	15.32	0.00	99.83	58.98
10	59.53	13.93	0.00	132.36	60.40
11	62.66	13.20	0.32	127.62	63.92
12	51.64	15.30	0.32	99.26	51.11
13	54.27	14.18	1.71	99.26	55.14
14	57.26	14.51	0.36	113.16	59.41
15	55.13	14.27	0.36	96.14	57.41
16	54.12	14.34	0.34	96.14	54.23
17	50.80	15.56	1.70	124.03	50.69
18	48.85	13.46	0.27	90.88	49.25
19	49.24	12.15	3.60	81.36	50.66
20	51.56	9.75	20.52	78.66	52.30
21	49.16	9.78	17.88	78.61	49.33
22	46.30	12.58	1.59	78.74	45.26
23	39.17	14.34	0.00	78.42	40.41

**Table 2:** Results of the Hydro-based GenCo’s Self-Scheduling According to Various Forecast Methods for 24 weeks

24 Weeks	Profit	Profit Loss	ELI	PFDI	MAE
Actual	9815726	_-_	-	-	-
Naïve	9513169	302557	0.0308	1.6576	9.3066
SARIMA	9576815	238911	0.0243	1.3089	8.3289
ANN	9594006	221721	0.0226	1.2147	**6.3774**
LSTM	9589966	225760	0.0230	1.2369	6.5489
GRU	9584191	231536	0.0236	1.2685	6.4586
LSTM–GRU	9596195	219532	0.0224	1.2027	6.4472
ANN–GRU	9591269	224457	0.0229	1.2297	6.3929
ANN–LSTM	**9599316**	**216410**	**0.0220**	**1.1856**	6.3851
ANN–LSTM–GRU	9597754	217972	0.0222	1.1942	6.4018
