# Supplementary material for: The Financial Effect of the Electricity Price Forecasts’ Inaccuracy on a Hydro-Based Generation Company
Source: Energies (Basel). Author manuscript; Available in PMC 2021 Jun 15. (PMC7610985; doi:10.3390/en11082093)
Supplement: Appendix [file EMS127017-supplement-Appendix.pdf]

Appendix A. Mathematical Model

We define the set values, parameters and decision variables in Tables A1– A3 correspondingly.

Table A1. Set values.

Set Values	
M	Plants of the hydro generating company
T	Time periods (hour) {1,.., T}
K	Performance curves {1,..,K}
L	Set of blocks relating to the performance curve {1,.., L}
ω_i	Upstream reservoirs of plant i

Table A2. Parameters.

Parameters	
M	Conversion factor (3.6 × 10^{-3} Hm^3s/m^3h)
λ_t	Forecasted price of energy in period t ($/MWh)
P_i	Capacity of plant i (MW)
PO_{ki}	Minimum power output of plant i for performance curve k (MW)
SU_i	Start-up cost of plant i
U_i^{min}	Minimum water discharge of plant i (m^3h/s)
U_{li}	Maximum water discharge of block l of plant i (m^3/s)
W_{it}	Forecasted natural water inflow of the reservoir associated to plant i in period t (Hm^3/h)
$X0_i$	Initial water content of the reservoir associated to plant i (Hm^3)
XF_i	Final water content of the reservoir associated to plant i (Hm^3)
XL_i	Lower bound of the water content pertaining to the reservoir of plant i (Hm^3)
XU_{ki}	Upper bound of the water content to the kth performance curve of plant i (Hm^3)
β_{lki}	The slope of the lth block of the kth performance curve of plant i ($MW/m^3/s$)
Γ_{ij}	Time delay between reservoir of plant i and plant j (h)
s_i^{max}	Maximum spillage of the reservoir associated to plant i (m^3/s)

Table A3. Decision Variables.

Decision Variables	
d_{kit}	0/1 variable used for the discretization of the performance curve k
v_{it}	0/1 variable which is equal to 1 if plant i is on-line in period t
y_{it}	0/1 variable which is equal to 1 if plant i is started-up at the beginning of period t
z_{it}	0/1 variable which is equal to 1 if plant i is shut-down at the beginning of period t
w_{lit}	0/1 variable which is equal to 1 if water discharged i has exceeded block l in period t
p_{it}	Power output of plant i in period t (MW)
s_{it}	Spillage of the reservoir associated to plant i in period t (m^3/s)
u_{it}	Water discharge of plant i in period t (m^3/s)
μ_{lit}	Water discharge of block l of plant i in period t (m^3/s)
x_{it}	Water content of the reservoir associated to plant i in period t (Hm^3)

The objective function (A1) maximizes the total profit of the hydro GenCo. In this equation, total profit equals to total revenue coming from the sales of the produced energy minus total start-up costs of the plants. Constraint sets (A2)–(A5) determine water volume of the plants according to the performance curves. Each performance curve is active at the predetermined intervals of the water volume based on the discretization of the non-linear functions. Constraints (A6) and (A7) calculate power generation of a plant according to the minimum power output associated with active performance curve, the total discharged water of the blocks and the power output capacity of the plant. Constraint set (A8) is the water balance equation. The total amount of the water content, spillage and discharged water from a plant in a period is equal to the total amount of the previous water content, natural water inflow, spillage and the discharged water amounts of the upstream reservoirs associated with the plant. Constraint set (A9) determines the discharged water amount of a plant based on water discharge of the reservoir blocks and minimum water discharge. Constraint sets (A10)–(A13) determine

the discharged water by the reservoir blocks of a plant. Constraint set (A14) ensures the spillage of a plant does not exceed the maximum spillage amount. Constraint sets (A15) and (A16) ensure the initial and final amount of the water content equals to the predetermined amounts. Constraint set (A17) is the logical statements to arrange the start-up and shut down status of the plants. Constraint sets (A18)–(A21) show the type of the variables and sign restrictions.

$$\text{Maximize} \sum_t \sum_i \lambda_t po_{it} - \sum_t \sum_i SU_i y_{it} \tag{A1}$$

s.t.

$$x_{it} \leq XU_{ki} d_{k-1,it} + \sum_{k=2}^{K} XU_{ki} d_{k-2,it} - d_{k-1,it} \quad \forall i \in I, \forall k \in K, \forall t \in T \tag{A2}$$

$$x_{it} \geq XU_{k-1i} d_{k-1,it} + \sum_{k=3}^{K} XU_{ki} d_{k-2,it} - d_{k-1,it} \quad \forall i \in I, \forall k \in K, \forall t \in T \tag{A3}$$

$$x_{it} \geq XL_i \quad \forall i \in I, \forall k \in K, \forall t \in T \tag{A4}$$

$$d_{1it} \geq d_{2it} \geq \ldots \geq d_{kit} \quad \forall i \in I, \forall k \in K, \forall t \in T \tag{A5}$$

$$p_{it} - P0_{ki} v_{it} - \sum_l \mu_{lit} \beta_{lki} - P_i[(k-1) - \sum_{n=1}^{k-1} d_{kit} + \sum_{n=k}^{K-1} d_{kit}] \leq 0 \quad \forall i \in I, \forall k \in K, \forall t \in T \tag{A6}$$

$$p_{it} - P0_{ki} v_{it} - \sum_l \mu_{lit} \beta_{lki} - P_i[(k-1) - \sum_{n=1}^{k-1} d_{kit} + \sum_{n=k}^{K-1} d_{kit}] \geq 0 \quad \forall i \in I, \forall k \in K, \forall t \in T \tag{A7}$$

$$x_{it} = x_{it-1} + W_{it} - M[u_{it} + s_{it}] + M[u_{it} + s_{it}] + M \sum_{i \in \Omega_i} [u_{i,t-\Gamma_{ij}} + s_{i,t-\Gamma_{ij}}] \quad \forall i \in I, \forall t \in T \tag{A8}$$

$$u_{it} = \sum_l \mu_{lit} + U_i^{min} v_{ti} \quad \forall i \in I, \forall t \in T \tag{A9}$$

$$\mu_{1it} \leq U_{1i} v_{it} \quad \forall i \in I, \forall t \in T \tag{A10}$$

$$\mu_{1it} \geq U_{1i} w_{1it} \quad \forall i \in I, \forall t \in T \tag{A11}$$

$$\mu_{lit} \leq U_{li} w_{l-1,it} \quad \forall i \in I, \forall t \in T, \forall l \in L \tag{A12}$$

$$\mu_{lit} \geq U_{li} w_{lit} \quad \forall i \in I, \forall t \in T, \forall l \in L \tag{A13}$$

$$s_{it} \leq s_i^{max} \quad \forall i \in I, \forall t \in T \tag{A14}$$

$$x_{i0} = X0_i \quad \forall i \in I \tag{A15}$$

$$x_{iT} = XF_i \quad \forall i \in I \tag{A16}$$

$$y_{it} - z_{it} = v_{it} - v_{i,t-1} \quad \forall i \in I, \forall t \in T \tag{A17}$$

$$d_{kit}, v_{it}, y_{it}, z_{it} \in 0,1 \quad \forall i \in I, \forall t \in T \tag{A18}$$

$$w_{lit} \in 0,1 \quad \forall i \in I, \forall t \in T, \forall l \in L \tag{A19}$$

$$p_{it}, s_{it}, u_{it}, x_{it} \geq 0 \quad \forall i \in I, \forall t \in T \tag{A20}$$

$$\mu_{lit} \geq 0 \quad \forall i \in I, \forall t \in T, \forall l \in L \tag{A21}$$
